# Meeting the global protein supply requirements of a growing and ageing population

**DOI:** 10.1007/s00394-024-03358-2

**Published:** 2024-03-02

**Authors:** Kieran Smith, Anthony W Watson, Marta Lonnie, Wouter M Peeters, Dennis Oonincx, Niki Tsoutsoura, Genis Simon-Miquel, Kamil Szepe, Noriane Cochetel, Alice G Pearson, Oliver C Witard, Andrew M Salter, Malcom Bennett, Bernard M. Corfe

**Affiliations:** 1 Oxford Centre for Diabetes, Endocrinology and Metabolism, Churchill Hospital, University of Oxford, Oxford, UK; 2 School of Biomedical, Nutritional and Sport Sciences, Newcastle University, Newcastle upon Tyne, UK; 3 Faculty of Medical Sciences, Human Nutrition and Exercise Research Centre, Population Health Sciences Institute, Newcastle University, Newcastle upon Tyne, UK; 4 The Rowett Institute, School of Medicine, Medical Sciences and Nutrition, University of Aberdeen, Aberdeen, UK; 5 Department of Human Nutrition, University of Warmia and Mazury in Olsztyn, Sloneczna 45F, Olsztyn, 10-718 Poland; 6 Animal Nutrition Group, Wageningen University & Research, Wageningen, The Netherlands; 7 Division of Food, Nutrition & Dietetics and Future Food Beacon, School of Biosciences, University of Nottingham, Nottingham, UK; 8 Leibniz Centre for Agricultural Landscape Research (ZALF), Müncheberg, Germany; 9 Department of Sport and Exercise Sciences, Durham University, Durham, UK; 10 Centre for Human & Applied Physiological Sciences, King’s College London, London, UK; 11 School of Life Sciences and Food Systems Institute, University of Nottingham, Nottingham, Nottingham, UK

**Keywords:** Protein, Protein quality, Global patterns, Life course, Emerging proteins, Sustainability, Novel strategies

## Abstract

Human dietary patterns are a major cause of environmental transformation, with agriculture occupying ~ 50% of global land space, while food production itself is responsible for ~ 30% of all greenhouse gas emissions and 70% of freshwater use. Furthermore, the global population is also growing, such that by 2050, it is estimated to exceed ~ 9 billion. While most of this expansion in population is expected to occur in developing countries, in high-income countries there are also predicted changes in demographics, with major increases in the number of older people. There is a growing consensus that older people have a greater requirement for protein. With a larger and older population, global needs for protein are set to increase. This paper summarises the conclusions from a Rank Prize funded colloquium evaluating novel strategies to meet this increasing global protein need.

## Global patterns of protein consumption and supply

**Fig. 1:**
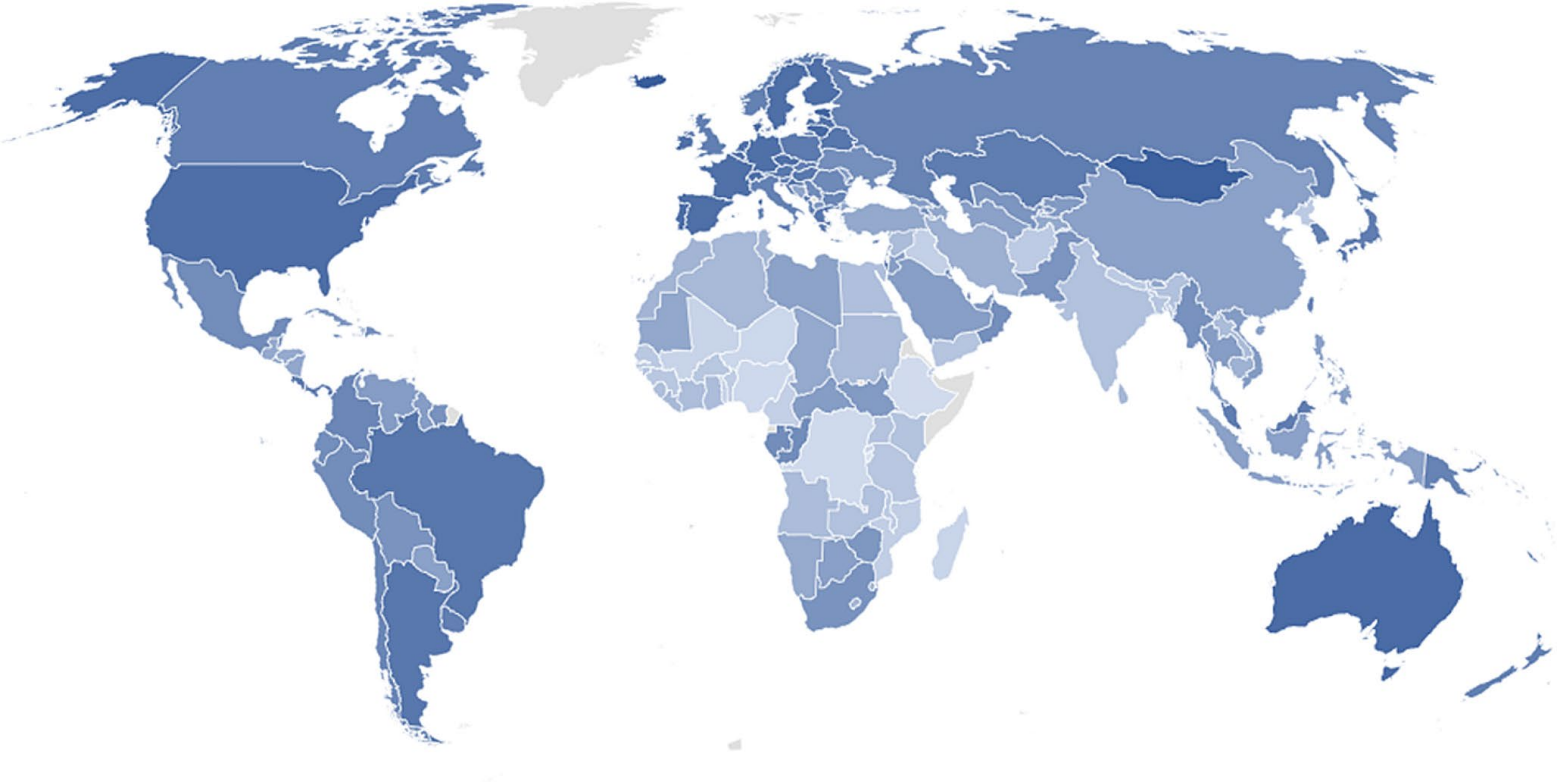
World heat map detailing the amount of animal protein consumed per capita per day. Protein consumption increases with depth of colour. Data range 6.74-77.13 g/day of protein. Grey indicates that data were not available for the country. Data taken from the 2020 FAOSTAT database (available from: https://www.fao.org/faostat)

Dietary proteins are derived from animal-, plant-, fungal- and bacterial-based foods. Globally, vegetal sources of protein dominate the protein supply (~ 60%) with remaining contributions from animal-derived proteins (meat, poultry, dairy and fish) [1], although their relevant contribution to the overall protein intake at the population level differs between global regions (Fig. 1). For example, countries in Africa, Asia, and South America have a long history of using plant-based products and insects in their cuisine, rather than vertebrate-derived animal products [2]. In West Africa and Southern India, where the prevalence of protein malnutrition in children and infants is high [3], rice and millet are consumed extensively and make up a large share of the food basket. In contrast, populations in wealthier regions form less than one fifth of the global population, but consume over one third of global animal proteins [4]. For instance, ~ 50% of protein intake in the United States is derived from animal products [5], where *per capita* meat consumption is three times the global average [6]. Similarly, although the intake of red meat and processed meats is declining in the United Kingdom [7], animal-based protein remains the major source of dietary protein [1].

Urbanisation and economic development are both associated with increased global consumption of animal-derived protein. Specifically, more poultry, pork and a larger fraction of processed meat are being consumed [8]. Evident over the coming decades will be a rapid demographic change with more than two thirds of the world’s population becoming urbanised and with an increase in average age. Together, these dual factors will drive an increase in the global protein requirement [9].

## Protein intake needs across the life course

Protein is a critical nutrient to support growth and development, and for the maintenance of musculoskeletal health throughout life. Animal-based protein foods are a rich source of energy, essential amino acids, and other essential nutrients (including iron, zinc, and vitamin B_12_) that can be difficult to obtain solely from plant sources [10, 11]. This nutritional profile can be essential for a large proportion of the global population who have limited access to foods, and where stunted child growth due to protein inadequacy remains a debilitating issue [8]. On the other hand, prospective studies in Western populations suggest that reducing meat intake, or replacing animal proteins with plant-derived proteins, may improve metabolic health and reduce the risk of premature mortality [12], cardiovascular disease [13] and type 2 diabetes [14]. Moreover, processed meat is classified as a carcinogen to humans [15], and the excessive consumption of red and processed meats are associated with colorectal cancer [16]. However, it is important to note that these associations are between the high consumption of animal products rather than dietary proteins per se, and disease risk is often confounded by other unfavourable lifestyle factors (obesity, inactivity, smoking, or excessive alcohol consumption) [12]. Indeed, several interventions focussed on increasing intake of dietary protein suggest that this dietary strategy can be effective in managing obesity and type 2 diabetes [17–19].

Dietary guidelines recognise the increased protein requirement during pregnancy, lactation and growth in infants and children. However, these guidelines tend to be consistent across the adult lifespan and have the goal of maintaining a constant state of skeletal muscle protein homeostasis. In the United Kingdom, the national recommended daily allowance (RDA) for protein ingestion in adults is 0.75 g of protein per kg of body mass per day (g/kg/day). However, this guideline should be viewed as the minimum requirement to prevent net nitrogen loss rather than a universal guideline or recommendation that is sufficient to meet the needs of different adult populations. In adults over 40 years old, there is progressive loss of muscle mass at a rate of around 8% per decade from 40 to 70 and 15% per decade thereafter [20]. This progressive age-related decline in muscle mass can be exacerbated by multiple clinical factors (obesity, malnutrition), low activity levels, and insufficient protein and micronutrient intake [21]. Furthermore, the protein requirements to achieve skeletal muscle homeostasis may be higher in older adults [22, 23] and adults living with morbidities [24]. This notion is supported by three lines of evidence. First, several well controlled, albeit short-term, studies reported that the protein RDA was inadequate for maintaining whole-body nitrogen equilibrium (as the standard method to determine protein requirements) in older adults [25–27], specifically hospitalised patients [28]. Second, a retrospective study estimated nitrogen equilibrium was achieved at a protein intake of 0.91 g/kg/day [29], which exceeds the current RDA by 15%. Third, studies using the more contemporary indicator amino acid oxidation (IAAO) technique have reported increased protein requirements in older adults compared with younger counterparts [30]. Collectively, these data suggest that a protein RDA of 0.75 g/kg/day underestimates the minimal protein requirement for older adults. This understanding has resulted in calls for higher intake requirements for older adults of between 1.2 and 1.7 g/kg/day [23, 24]. However, these higher protein recommendations are broadly based on an omnivorous diet and do not account for protein quality [31]. The achievement of equivalent efficacy through plant-based sources alone will typically require higher levels of intake [32]; although combining plant proteins can produce a complimentary amino acid profile, thereby circumventing amino acid deficiencies [33].

Despite guidelines for elevated protein requirements, many older adults are not consuming sufficient dietary protein. Within North America, ~ 40% of older adults are reported to consume less protein than the RDA [34, 35]. Similarly, although protein intake over the last decade has remained relatively constant across the adult general population (> 19 years) within the United Kingdom (at ~ 76 g per day [~ 17% total energy intake]), habitual dietary protein intake declines as people age; i.e. protein intake is 11–19% lower in adults > 75 years compared to adults aged 64 years or less [36]. A similar observation is seen in Dutch and Italian older adults, with 21.5–35% of older adults consuming less than the RDA of 0.7–0.8 g/kg/day [37, 38]. Interestingly, the relative contribution of animal proteins to overall protein intake is lower in older individuals consuming less gross dietary protein [39]; thus, deficits in dietary protein intake may be further exacerbated by lower quality protein intake [31]. It is interesting to note that the increased intake of animal-derived proteins was recently shown to be inversely associated with mortality in Italian community-dwelling older adults over a 20-year observation period [40]. This may be due to the positive association between the greater intake of sources of higher protein quality and physical function and muscle strength [41], which in turn has been shown to be protective against premature mortality in older adults [42]. However, many older adults are under the perception that they currently consume sufficient dietary protein and are sceptical about increasing their dietary protein intake for health and well-being purposes [43]. This stance is of particular concern for elderly adults with poor “protein knowledge” who are more likely to have reduced physical function [44].

## Protein quality

Most dietary protein recommendations are based on the assumption of high-quality protein intake. For example, the UK Dietary Reference Values specifically states that protein recommendations are based on the assumption that the ingested protein is of high quality; in other words, the essential amino acid composition in food proteins is close to the human body’s need [45]. This means that proteins have an appropriate indispensable amino acid (IAA) composition and digestibility profile to meet human requirements. Nevertheless, the quality of protein from different protein sources can vary substantially. In general, plant proteins tend to be of lower protein quality, compared to animal-derived proteins. This observation is attributed to an incomplete amino acid composition, i.e. lysine and methionine contents are typically lower in plant proteins than animal proteins [46], or reduced digestibility due to the presence of anti-nutritional factors, interactions with other food components such as fibre, the structure of the protein itself, or a combination of such factors [47]. However, the consumption of a mixed-diet, including protein from both plant- and animal-sources, are of high protein quality due to the complementary amino acid profiles [48]. Potentially complementary proteins must be consumed concurrently to have synergistic effects- an excess of one essential amino acid that is limited in one protein would provide no benefit to muscle protein synthesis if it is elevated at a different time from when the other essential amino acids are absorbed [49].

The determination of protein quality is challenging, and in vivo assessments are expensive and invasive. The FAO suggests that assessments of protein quality should be based on the digestible indispensable amino acid score (DIAAS), which is defined as the amount of IAA in a gram of protein divided by the amount of the same amino acid in a gram of a reference protein [50]. The DIAAS protein quality score is based on digestibility at the end of the ileum, rather than relying on faecal determination, which is impacted by endogenous and microbial proteins [51]. For a mixed-diet, a DIAAS score of 1.0 or > 1.0 indicates that the dietary protein is fully utilised (accounting for ileal digestibility), whereas values below 1.0 demonstrate less complete utilisation of the ingest protein [52]. The latter indicates that more protein would have to be ingested to meet the physiological requirements for essential amino acids [49]. In vivo measurements of the ileal digestibility of individual amino acids in humans are still relatively sparse and, as such, are dependent on measurements in other animal species such as pigs or rats. Recent developments in in vitro digestion modelling systems should enhance our ability to screen the quality of novel protein sources and the potential impact of cooking and processing techniques [53].

Recognising proficient protein sources in the diet is challenging for consumers. Currently, this information is limited to the amount of protein per 100 g of product or per portion, or a nutrition claim that a product is a protein source if > 12% of energy is derived from dietary protein. As a result, food labelling may favour animal-based products because these tend to contain more protein per 100 g of product. Assessing protein quality on the single protein basis also omits the fact that foods are consumed as part of a meal. As described, combining plant proteins can help to achieve the required levels of high-quality protein, compared to an animal-based meal or a single product [33].

## Tensions between human health and planetary status within the context of a growing and ageing population

**Fig. 2:**
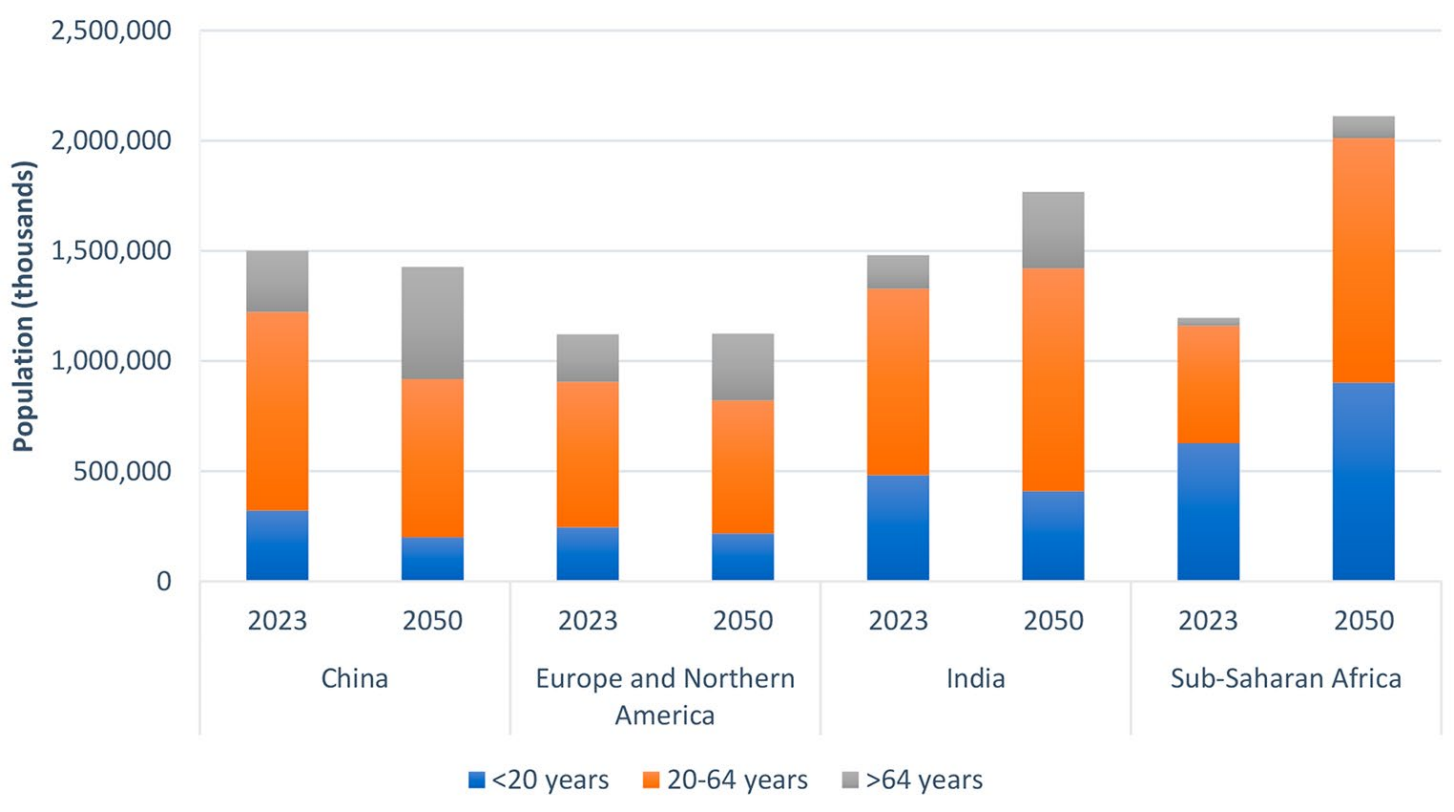
Current (2023) and predicted (2050) populations of selected countries/regions of the world. Data represents medium variant taken from United Nations Population Division, Department of Economic and Social Affairs (https://population.un.org/wpp/Download/Standard/MostUsed/)

The increased protein requirement of a growing and ageing global population has significant implications for planetary status. Human dietary patterns are the largest cause of environmental transformation with agriculture occupying ~ 50% of global land space, while food production itself is responsible for ~ 30% of all greenhouse gas emissions and 70% of freshwater use [54, 55]. It has contributed to planetary boundaries that define a safe operating space for humanity on a stable Earth system to be exceeded and poses the risk of destabilising the ecosystem on which populations depend on [56]. Should the global population reach 9 billion with ~ 30% of the population classified as older adults, it is estimated that global protein supply requirements will be elevated by 20% [57]. However, it is also important to recognise the potential impact of different global demographics. In China, Europe and North America, the total population is predicted to stabilise, or even decrease, but with a greater proportion of older people. In contrast, India will see a relatively modest increase in adult population, while the total population of Sub-Saharan Africa, where protein energy malnutrition is still relatively common, will almost double (Fig. 2).

The increased demand for dietary proteins, particularly animal-proteins [58], will have a major impact on the global food system and land use [59]. Despite providing only 37% of protein supply, animal products (meat, aquaculture, eggs and dairy) use 83% of the world’s farmland and contribute to ~ 58% of food production related emissions [6]. The rearing of livestock is also one of the main ways by which humans directly affect the environment: forests are cut down to generate pastures and arable land for animal feed, the feed-to-product ratio is vastly unbalanced, and the production of livestock are major greenhouse gas emitters [8]. On a gross protein basis, animal-derived proteins are major emitters of greenhouse gas emissions and freshwater use [6]. Yet, when expressed per kg of digestible lysine (i.e., protein quality) or as a per 100 g of food for DIAAS, the environmental footprint of several animal-derived proteins (including pork, egg, and milk production) is similar to that of plant proteins [52, 60]. Moreover, as shown by modelling analyses conducted in the United States, although agricultural-associated greenhouse gas emissions would be reduced by 28% by removing animal-derived proteins from the food system, this would come at a cost of severe nutrient deficiencies (e.g., vitamins A and B_12_, arachidonic, eicosapentaenoic, and docosahexaenoic fatty acids) and an increase in overall energy (kcal) consumption [5]. If advocacy for plant-based protein sources were universally adopted, it is estimated that this would increase the global supply of protein requirements by 50% [32]. These estimates are stark figures that show the goals of maintaining human health at a population level and in an ageing population will place considerable stress on the global supply chain and environment. Hence, it is critical that UN Sustainable Development Goals are not in conflict and are open to exploring novel approaches to meeting global protein requirements alongside tackling associated challenges in achieving these aims.

## Novel strategies (and their risks) for meeting elevated protein requirements

Increasing the production of animal-derived proteins, using current farming practices, does not offer a sustainable solution to meeting global needs. Livestock utilises human–edible protein inefficiently, consumes a large proportion of global land and water resources and is associated with a significant proportion of global greenhouse gas emissions [6]. To a certain extent, some of these adverse effects could be mitigated by changes in farming practices, including a shift to using alternative protein sources in livestock feed [61]. However, there is growing recognition that if we are to sustainably provide sufficient protein for the global population, we need to diversify available sources of dietary protein and become less reliant on animal-derived protein. This trend is already occurring in countries such as the United States and United Kingdom, where beef consumption reduced modestly (-5.7 g/day [11% point reduction]) between 2001 and 2018 in adults aged under 60 years of age [7, 62].

### Plant-based sources

In order to meet future protein requirements, it is likely that in addition to increasing consumption of existing plant sources we also need to develop novel protein sources that are more sustainable and resistant to the inevitable adverse effects observed due to climate change. The nature of these changes is likely to vary considerably depending on environmental, sociological and economic factors associated with different populations. In Western Societies there is increased interest in, and acceptance of, plant-based meat alternatives, such as vegetable-based burgers. Indeed, evidence from a Dutch cohort has suggested that some individuals would consider or be willing to consume one less meal containing meat per week [63]. In such a situation, the lower quality of plant-based proteins may require greater intakes compared to dietary proteins derived from animals [32], but this dietary pattern may be offset by consuming plant-protein blends or mycoproteins (from fungi) that offer a better balance in terms of amino acid profile and digestibility [33]. The situation in lower-income countries is very different. Many such populations are highly dependent on a single source of plant protein, often from cereals, that are often deficient in specific amino acids and/or poorly digestible. For example, in the poorest households in Malawi, almost 80% of protein intake is from cereal crops, predominantly maize [64]. According to a database of ileal digestibility and DIAAS values of world foods [65], it was calculated that 63% of such households were at risk of deficiency of the IAA, lysine. While biofortification of crops could potentially improve the quality of protein, e.g. Quality Protein Maize [66], diversification of protein sources, including additional animal protein, is likely to be required to meet the needs of such growing populations.

### Vertebrate-derived protein

There is interest in complementing vertebrate-derived protein with alternative sources of animal protein, particularly insects either in whole form, or incorporated into flour. Edible insects are rich in fat and micronutrients [67], and serve as a highly digestible and functional protein [68]. Moreover, insects can be reared on organic side-streams, leading to lower greenhouse gas emissions, water use and land use compared to conventional animal proteins [69, 70]. Whilst > 2000 insect species exist that are suitable for human consumption, the inclusion of insects in the human diet within Westernised cultures is perceived as undesirable and associated with food taboos [71].

Cultured meat and cellular agriculture produced using tissue engineering and synthetic biological approaches to manufacture meat-resembling products represents another novel area to meet the global protein requirements without the drawbacks of conventional animal agriculture [72]. These products offer the advantages of sustainability and have the potential to replace livestock, but considerable work is still required to make them economically competitive.

### Valorisation of waste

Opening novel routes to protein production also offers opportunities to valorise waste and reduce other environmental impacts. In the European Union, over 1.4 billion tons of manure is generated and considered a waste product with grave environmental effects, such as soil acidification, air and water pollution, and biodiversity loss [73]. Work has shown that certain insect species, such as the housefly and the black soldier fly, develop effectively on manure and indeed can reduce the manure to a third of its original mass [74]. The microbes in the manure, including those pathogenic to humans, are used as a nutrient source by the larvae, thereby decreasing their presence [75]. Moreover, a proportion of the ammonia present in manure is converted to body protein, possibly via microbial conversions [76]. The resulting larvae can then be fractionated and used as petfood, as proteinaceous feed ingredients for pigs and poultry, and their fat used as biofuel. In this way, feed-food competition is reduced and contributes to meeting protein requirements. Similarly, proteins derived from animal by-products, e.g. keratin extracted from poultry feathers and coarse wool, may offer another opportunity to valorise an abundant waste product into an effective and usable protein source [77, 78].

### Considerations

It is important to recognise that novel protein sources may carry risk to some people. In the United Kingdom, it is estimated that ~ 0.5% of adults and ~ 2% of children suffer with peanut allergy [79]. Peanuts are closely related to legumes which occupy an increasing share of the alternative protein market, in particular pea protein [80]. Peanut-allergic consumers may experience “cross-reactive allergy”, where IgE antibodies against peanut proteins react with proteins in pea. Currently, there is no requirement for labelling pea as an allergen on food labels in the same way that other allergenic legumes (i.e., peanut, soy, lupin) must be labelled. Indeed, peas have been widely consumed in the United Kingdom; however, it is the novel format (higher doses of concentrated pea protein) that has revealed this issue to the clinical allergology community. Plant-derived proteins also contain anti-nutritional factors, including lectins, phytic acid, and enzyme inhibitors, that may impair the digestion and absorption of other nutrients [47]. This reduction in postprandial amino acid availability may be remedied during food preparation and cooking processes, but in turn cultural adaptation to new protein sources will be needed.

Animal proteins play an essential role in addressing macro- and micronutrient deficiencies in many regions of the world [10]. Livestock industries are also an important component of agricultural economies and provide livelihoods for up to 1 billion poor smallholder farmers in the developing world, thereby offering pathways out of poverty [8]. In areas unsuitable for crop cultivation, livestock is the only option for rural livelihoods. As such, the transformation in the food system and integration of novel protein-sources needs to occur alongside the focus on rural development and poverty reduction.

## Conclusions

Ensuring adequate dietary protein intake for a growing and ageing global population poses a major challenge. In many parts of the world where populations are predicted to grow most rapidly, protein deficiency is already common. In high-income countries the proportion of older adults with increased protein needs continues to grow. This could be exacerbated in developing countries where there is an increase in the general and ageing population and an increase in urbanisation. Sustainably meeting the protein requirements of future generations will require dramatic changes in food systems. Accordingly, considerable scaling and refinement of production methods associated with alternative protein commodities will be critical for the mass delivery to market and the shaping of a new protein economy. Consumer acceptability of non-traditional protein sources requires careful consideration alongside improvements in the functionality of novel sources. Major shifts in policy and investments are fundamental to transitioning the current global food system from a threat to a solution space for human and planetary health. At a policy level, it is vital to consider current consumer knowledge and preferences in order to effectively communicate the net benefits of increasing the amount and diversity of protein sources in the diet. To secure consumer acceptance of novel foods, and trust in their safety, comprehensive and rational allergenicity risk assessment is vital. There is an urgent need for further research to ensure affordable, healthy and sustainable protein sources to meet the needs of future generations across the world.
